# Post-treatment of Fungal Biomass to Enhance Pigment Production

**DOI:** 10.1007/s12010-019-02961-y

**Published:** 2019-04-08

**Authors:** Rebecca Gmoser, Jorge A. Ferreira, Mohammad J. Taherzadeh, Patrik R. Lennartsson

**Affiliations:** Swedish Centre for Resource Recovery, University of Borås, Allégatan 1, 503 32 Borås, Sweden

**Keywords:** Pigments, *Neurospora intermedia*, Carotenoids, Edible filamentous fungi, Post-treatment

## Abstract

A new post-treatment method of fungal biomass after fermentation is revealed. The post-treatment strategy was utilized to produce pigments as an additional valuable metabolite. Post-treatment included incubation at 95% relative humidity where the effects of harvesting time, light, and temperature were studied. Pigment-producing edible filamentous fungus *Neurospora intermedia* cultivated on ethanol plant residuals produced 4 g/L ethanol and 5 g/L fungal biomass. Harvesting the pale biomass after 48 h submerged cultivation compared to 24 h or 72 h increased pigmentation in the post-treatment step with 35% and 48%, respectively. The highest pigment content produced, 1.4 mg/g dry fungal biomass, was obtained from washed biomass treated in light at 35 °C whereof the major impact on pigmentation was from washed biomass. Moreover, post-treated biomass contained 50% (*w*/*w*) crude protein. The post-treatment strategy successfully adds pigments to pre-obtained biomass. The pigmented fungal biomass can be considered for animal feed applications for domestic animals.

## Introduction

For a long time, filamentous fungi have been used for the industrial production of commercially relevant products, including enzymes, antibiotics, feed products, and many others [1]. The biorefinery concept, i.e., converting biomass into a spectrum of marketable products and energy [2], is anticipated to improve bioprocesses. Therefore, research towards the diversification of established and future facilities for the production of numerous novel and valuable products as well as by-products through fermentation is a hot topic. Filamentous fungi are being investigated as core biocatalysts in biorefineries, and are useful in creating new sustainable products. Some edible ascomycetes and zygomycetes fungi have proven to be excellent ethanol producers able to consume pentose sugars. Besides, the use of food-grade filamentous fungi to obtain nutritional rich biomass and other value-added products is highly interesting for the food, feed, cosmetic, and pharmaceutical industries [3]. These fungi provide alternative natural sources of products with low environmental impact partly due to their ability to grow on and produce industrial metabolites from different waste streams.

Submerged fermentation (SmF) on waste streams promotes a high biomass production rate since the free-flowing liquid substrate is easy to utilize by the microorganisms [4, 5]. However, efficient production of several secondary metabolites may be hampered by submerged fermentation. The reason is that their production appears to be triggered by reduced water and nutrient supply, association of microbial mycelia with a solid substrate or with an inert support, low agitation, excess oxygen supply, and spore formation [5, 6]. The shift from the biosynthesis of primary to secondary metabolites occurs in microorganisms in order to preserve energy sources and essential metabolites for more favorable growth conditions [7]. A fast growth rate and a short fermentation process utilizing low-cost substrates are important strategies for industries to obtain a competitive production of demanding metabolites [8]. One of these secondary metabolites are pigments.

Natural sources of pigments are being revitalized, inter alia due to the increased environmental and health awareness from the consumer side. Industries, especially in the food, pharmaceutical, cosmetic, and feed market are thus searching for alternative sources of natural pigments. In the last decades, the global market for natural carotenoids has increased (annual growth rate of 3.9%) partly due to their proven health effects, as well as their use as additives in the food, feed, and cosmetic industry [9]. Thus, these yellowish-orange pigments provide an attractive platform for future research [8]. The wide selection of pigment-producing edible strains of filamentous fungi able to grow on residuals might be an effective and sustainable way to meet the marked need [1, 10]. Low-cost substrates are crucial in order to achieve cost-effective and environmentally friendly production facilities. One such low-cost substrate is thin stillage, of which 6–7 L is produced per liter of ethanol in the typical dry mill ethanol production process [11]. Therefore, thin stillage was chosen as a low-cost substrate for pigment production in this study.

Generation of secondary metabolites such as pigments in the fungal biomass often occurs after fungal growth has ceased as a result of nutrient limitations and/or external stresses, as a protective mechanism [12]. For example, a fermentation medium with imbalanced nutrient content, especially in carbon and nitrogen, was reported to support the fungus *Neurospora crassa* to produce β-carotene in a mixture of 60% tapioca by-product and 40% tofu waste [13]. The edible filamentous fungus *Neurospora intermedia* used in this study has traditionally been used for preparation of the Indonesian food *oncom*, and has recently been reported as a potential biomass and ethanol producer from waste streams of the industrial process of ethanol production from agricultural grains [1, 14, 15]. *N. intermedia* is also able to produce biomass-containing carotenoids. Carotenoid production by the fungus has previously been reported to be influenced by a number of cultural and environmental factors such as light, low pH, solid support, changes in nutrition, and increased aeration supply [11, 12, 15]. Addition of penicillin [16, 17], up to 10 mM Mg^2+^, and 3 days incubation in dark condition followed by 4 days under blue light have been reported to stimulate *N. intermedia* carotenoid biosynthesis. A previous study on *N. crassa* also proposes a higher amount of pigments being produced in the conidia and in the conidia-bearing hypha. Conidia are only formed in the air; none are formed in submerged mycelia [18].

In an attempt to overcome today’s challenges regarding secondary metabolite production from filamentous fungi, an innovative post-treatment method of fungal biomass is presented in this work. The method comprises submerged cultivation to obtain a high amount of biomass, with post-treatment of the recovered biomass to stimulate synthesis of secondary metabolites. The edible filamentous fungus *N. intermedia* was used to determine the method’s potential to convert the by-product thin stillage into pigmented biomass. The effect of submerged fermentation time, influence of light, temperature, and washed or unwashed biomass as well as the biomass moisture content and thickness in the post-treatment were studied concerning pigment production. The process was also scaled up to 1000-L bioreactor to determine its robustness.

## Materials and Methods

### Substrate

A single batch of thin stillage was obtained from Lantmännen Agroetanol (Norrköping, Sweden), a dry-grind ethanol plant mostly based on wheat. The thin stillage was autoclaved for 30 min at 121 °C and stored at 4 °C prior to use.

### Fungal Strain

The edible filamentous ascomycete *Neurospora intermedia* CBS 131.92 (Centraalbureau voor Schimmelcultures, The Netherlands) was used in the current study. The fungus was maintained on potato dextrose agar (PDA) medium containing (in g/L) glucose 20, agar 15, and potato extract 4. The PDA plates were prepared via incubation for 3–5 days at 30 °C followed by storage at 4 °C. Spore solution was prepared by flooding each plate with 20 mL of distilled water, using a disposable plastic spreader to release the spores. All liquid cultures were inoculated with 10 mL/L of spore solution with a spore concentration of 5.7 (± 0.3) × 10^5^ spores/mL.

### Fungal Cultivation on Thin Stillage in Bioreactors

#### Bench Scale Bubble Column Bioreactors

Thin stillage was used as the basal screening medium. Fungal cultivation was carried out aerobically in two bench scale airlift bioreactors operated as bubble column bioreactors (Belach Bioteknik AB, Skogås, Sweden) with a working volume of 3.5 L. The bioreactor was made of transparent borosilicate glass. An inoculum of 70 mL of spore suspension was added to 3 L thin stillage at pH 5.2, adjusted by addition of 2 M NaOH. Cultivations were carried out at 35 °C with an aeration rate of 1.3 vvm (volume_air_/volume_media_/min) passing through a sintered stainless-steel air sparger with a pore size of 0.2 μm. Filtration of inlet air was achieved by using a PTFE membrane filter (0.1 μm pore size, Whatman, Florham Park, NJ, USA). In order to examine the effect of cultivation time on post-treatment pigment production, cultivation lasted for 24 or 48 h, while other parameters remained constant. Antifoam silicone snapsil FD10 (VWR International, USA) was used to avoid foaming. Samples (1.5 mL) were taken regularly from the fermentation broth and centrifuged at 10,000×*g* for 10 min, and the supernatant was kept at − 20 °C until analysis by HPLC. The biomass was recovered at the end of cultivation by pouring the thin stillage through a sieve. The moisture content was determined by drying 1 g of the harvested biomass in triplicate at 70 °C until constant weight. The remaining biomass was used for post-treatment. Half of the biomass was extensively washed with a continuous flow of distilled water until a clear effluent was obtained while the remaining biomass was kept unwashed.

#### Cultivation in 26-L Bioreactor

A 2-m-high, 15-cm-diameter bubble column bioreactor with a total volume of 26 L (Bioengineering, Switzerland) was used for scale-up with an aeration rate of 1 vvm. The reactor was in situ sterilized with direct injection of steam (121 °C, 30 min). Twenty liters of thin stillage supplemented with 45 μL/L antifoam was used as medium. Inoculation was prepared in two 0.5-L Erlenmeyer flasks containing 0.2 L thin stillage cultivated for 24 h in a water bath set at 35 °C and orbital shaking at 125 rpm (resulting in 7.7 ± 0.8 g/L biomass dry weight). The temperature in this bioreactor was kept at 35 °C. After 24 h, the biomass was recovered by pouring the thin stillage through a sieve and washing extensively with distilled water until a clear effluent was obtained.

#### Cultivation in 1-m^3^ Airlift Bioreactor

A 4-m-high, 0.65-m-diameter airlift reactor (Process & industriteknik AB, Kristianstad, Sweden) with 1.38 m^3^ total working volume and an internal tube (2.7 m high, 0.4 m diameter) containing the fermentation medium was pasteurized in situ with injection of steam (temperature of 70 °C was increased continuously for 1 h reaching 90 °C). A total working volume of 900 L synthetic medium was used as the basal screening medium, containing per liter 37 g sucrose and 5 g yeast. The fermentation medium was supplemented with (mM) 16 ZnCl, 2.7 MnCl, 1.3 CoCl_2_, and 1.3 CuSO_4_·5H_2_O as well as 30 mL antifoam and 400 mg antimicrobial solution (Fermasure, Dupont) supplemented at four different time intervals. The cultivation was carried out for 48 h at 35 °C, 1.0 vvm aeration, and pH 4.0 ± 0.5 (by addition of 2 M H_2_SO_4_). The inoculum was prepared in the 26-L bubble column reactor (working volume of 20 L) operated as described previously, containing a synthetic medium composed of 20 g/L glucose and 5 g/L yeast extract. Inoculation was carried out as previously described, but using 20 g/L glucose and 5 g/L yeast extract.

### Post-treatment

Fungal biomass from the submerged fermentation (82 ± 1.8% moisture content on a wet base) was spread on petri dishes with a thickness of 1 cm followed by incubation in a climatic test cabinet (NUVE test cabinet TK 120, Turkey) for 24 h at 95% Rh ± 1%. Two representative samples (taken from two different places in the biomass, where each sample contains biomass from the surface and bottom, were taken from each plate for pigment analysis. A full 2^4^-factorial experiment was designed to examine the effect of different factors including cultivation time (24 or 48 h), light (6000 lx ± 10%)/dark environment, incubation temperature (25 or 35 °C ± 0.1), and washed/unwashed biomass on pigment production. The model used to analyze the effect from the factors, the normal probability plot of the residuals, and the residuals versus the predicted pigment production from the model containing the identified factors showed a non-constant equality of variance and deviation from normality. The problem can be linked to the increased dispersion proportionally to the value, indicating the need for a variance-stabilizing transformation [19]. A log transformation (*λ* = 0) of the data (*y* = ln *y*) was used, resulting in a transformed model with homoscedastic and fairly well normally distributed residuals. The additive effects in the transformed model are converted into multiplicative effects for the original response variable. All the data obtained were analyzed using a general linear model analysis of variance (ANOVA). Factors were considered significant at a *p* value less than 0.05, and the effects of treatment combinations were analyzed using the Tukey multiple comparison test. Statistical analysis of the data was performed using the software package MINITAB® (version 17.1.0, Minitab Inc., State College, PA, USA). The experimental design resulted in eight different combinations for each light source with one replication of samples. In order to improve the method further, examination of important factors related to pigment production was designed as single-factor experiments (Table 1). For the effect of biomass harvesting time, the biomass samples were washed with distilled water and incubated in the climatic test cabinet for 24 h, keeping 95% Rh ± 1%, light, and 35 °C constant for all the experiments. The effect of different light exposure time (1 min, 1 h, and 24 h) on pigmentation was investigated by turning off the light in the climatic test cabinet while keeping the other parameters constant as for the biomass harvesting experiment. The effect of biomass moisture content (76, 83, 89, 93, or 96%) was analyzed with washed biomass taken after 48 h submerged fermentation before being incubated on petri dishes in the climatic test cabinet for 24 h, keeping 95% Rh ± 1%, light, and 35 °C constant.

**Table 1:** Factors and level of factors included in the full-factorial and single-factor experiments

Full-factorial experiment			
Light	Temp (°C)	Cultivation time (h)	Biomass
Light	25	24	Prewashed
Dark	35	48	Unwashed
Single-factor experiments			
Light exposure	Cultivation time (h)	Fungal biomass mc (%)	
1 min	24	76	
1 h	48	83	
24 h	72	89	
	96	93	
		96	

*mc* moisture content

Furthermore, the biomass thickness layer was investigated on washed biomass with a moisture content of ca. 80%, harvested after 48 h of submerged fermentation. Two glass beakers, one with aluminum foil-covered sides and one without, were filled with fungal biomass up to a thickness of ca. 2.5 cm and incubated for 24 h in the climatic test cabinet (95% Rh ± 1%, light, 35 °C). The experiments were carried out in duplicate.

These experiments were followed by two scale-up experiments in a 26-L bioreactor and a 1-m^3^ airlift reactor. The biomass from the 26-L bioreactor was washed with water and sieved to obtain a moisture content of ca 80% after which the biomass was transferred to the climatic test cabinet. Recovered biomass from the 1-m^3^ bioreactor was spread out on benches with a thickness of about 1.5 cm and kept for 24 h with light after the washing and sieving steps.

### Extraction and Estimation of *Neurospora intermedia* Orange Pigment

At the end of the post-treatment step, a representative sample of biomass was taken from each cultivation plate and washed with distilled water. The washed biomass (50 mg) was then extracted with 3 mL of 99% ethanol through sonication for 10 min, followed by centrifugation at 1200×g for 5 min. The supernatant was separated and the biomass was re-extracted with 99% ethyl acetate and centrifuged once more and then the supernatant was added to the first extraction solution. The extracted pigments in the ethanol/ethyl acetate solution were analyzed spectrophotometrically (Biochrom Ltd., Cambridge, England) at the maximum absorption of 466 nm, and the concentration was determined using a calibration curve for β-carotene dissolved in the same solution. After extraction, the biomass was dried at 70 °C for 24 h.

### Analytical Methods

Initial spore concentration was determined as previously described by Gmoser [15]. The liquid fractions from the thin stillage were analyzed using high-performance liquid chromatography (HPLC). A hydrogen-ion-based ion-exchange column (Aminex HPX-87H, Bio-Rad, Hercules, CA, USA) at 60 °C and 0.6 mL/min, 5 mM H_2_SO_4_ as eluent were used for analyses of glucose, other sugars, and ethanol. An ultraviolet (UV) absorbance detector (Waters 2487, Waters Corporation, Milford, MA, USA), operating at 210 nm wavelength, was used in series with a refractive index (RI) detector (Waters 2414). The harvested biomass was dried until constant weight in an oven at 70 °C and reported as biomass dry weight in grams per liter of stillage. The InKjel P digestor and the Behrotest S1 distiller (Behr Labor-Technik, Germany) were used to determine the total Kjeldahl nitrogen content in the biomass of *N. intermedia* before and after post-treatment. Crude protein content in the fungal biomass was estimated by multiplying the nitrogen content found in the sample by a nitrogen-to-protein factor of 6.25 [20]. All experiments were performed in duplicate, and the presented results are the mean of the observed values and the standard errors of the means.

## Results and Discussion

In this work, a method combining two steps was applied to attain high yields of pigmented biomass in a short time period. In the first step, submerged fermentation in bubble column reactors using thin stillage as substrate was performed to produce high amounts of fungal biomass and additional ethanol. Secondly, the harvested biomass was left in 95% Rh for 24 h exposed to air without any nutrition supplied, mimicking a stressed condition, to stimulate pigment production as a valuable secondary metabolite. The edible filamentous fungus *N. intermedia* successfully produced fungal biomass and ethanol in thin stillage after which pigments were produced. Protein content in the biomass remained rather constant. The results from the two-stage process are presented and discussed based on a set of assays performed studying different factors followed by a protein content analysis. Additionally, scale-up studies are presented.

### Main and Interaction Effects from Factors on Post-treatment Pigment Production

The effect of submerged cultivation time on pigment production during post-treatment was investigated by cultivating *N. intermedia* in bench scale (3.5 L) bubble column bioreactors. Post-treatment of the biomass by washed or unwashed biomass in light or dark condition at 25 or 35 °C and moisture content was investigated. The pigment concentration was measured after 24 h post-treatment in 95% Rh.

**Fig. 1:**
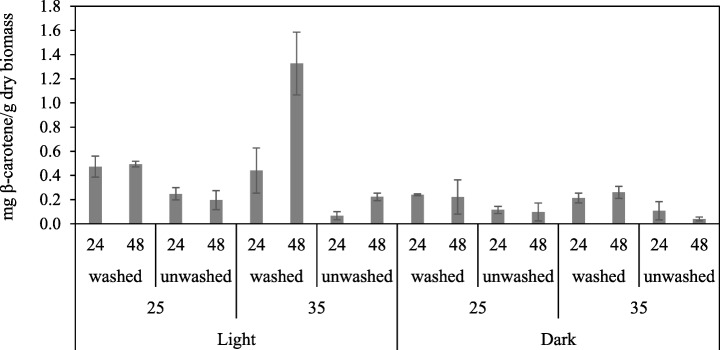
Individual mean values milligrams β-carotene/grams dry biomass for each group of combination. On the *x*-axis, 24 h and 48 h represent submerged fermentation time, washed or unwashed fungal biomass relates to a washing step or no washing with distilled water before post-treatment, 25 °C and 35 °C as well as light and dark represent the temperature and light source conditions during post-treatment of the fungal biomass in 95% relative humidity. Error bars represent the SD of the mean

The significant main and interaction effects are summarized in Table 2. The predicted main and interaction effect values on pigment production from the factors investigated are presented in Table 3. The Box-Cox transformation (*λ* = 0) generated an untransformed model with geometric mean values and multiplicative factorial effects. The transformed coefficients with standard errors and the fitted mean values for the untransformed model are presented in Table 3. The standard errors for the transformed coefficients are equal to the relative standard errors for the (multiplicative) untransformed coefficients. The specific combination between 48 h of submerged fermentation with post-treatment of washed biomass in light conditions at 35 °C gave the highest predicted pigment production (1.4 mg β-carotene/g dry fungal biomass). Pigment outcome from all the combinations of factors are presented in Fig. 1.

**Table 2:** General factorial regression (box-Cox transformation *λ* = 0) ANOVA—analysis of variances for transformed response (*y* = ln *x*), summarizing factors and interaction between factors comprises significant effect on pigment production. Factors were considered significant at *p* < 0.05. The model explains about 89% of the variability in the pigment production (*R*^2^ = 88.85). Symbol × refers to interaction between factors

Source	*T* value	*p* value
Constant	− 21.32	0.000
Light	5.7	0.000
Biomass	8.01	0.000
2-way interactions		
Light×submerged fermentation time	− 2.8	0.013
Temperature×biomass	− 2.61	0.019
Light×temperature	0.18	0.677
3-way interactions		
Light×temperature×submerged fermentation time	2.32	0.034
*S*	R-sq	R-sq (adj)
0.425462	88.85%	78.40%

**Table 3:** Transformed model coefficients with standard error (SE) values and fitted means for each significant main effect and interactions

Source	Fitted coefficients (transformed)	SE coefficient (transformed)	Fitted means (original)
Light	− 1.174	0.107	0.309
Dark	− 2.033	0.107	0.131
Washed	− 1.001	0.107	0.368
Unwashed	− 2.206	0.107	0.11
Light×24 h	− 1.446	0.152	0.235
Light×48 h	− 0.903	0.152	0.406
Dark×24 h	− 1.883	0.152	0.152
Dark×48 h	− 2.182	0.152	0.113
25 °C × washed	− 1.126	0.152	0.324
25 °C × unwashed	− 1.938	0.152	0.144
35 °C × washed	− 0.876	0.152	0.417
35 °C × unwashed	− 2.475	0.152	0.084
Light×25 °C × 24 h	− 1.081	0.215	0.339
Light×25 °C × 48 h	− 1.189	0.215	0.305
Light×35 °C × 24 h	− 1.812	0.215	0.163
Light×35 °C × 48 h	− 0.616	0.215	0.54
Dark×25 °C × 24 h	− 1.802	0.215	0.165
Dark×25 °C × 48 h	− 2.056	0.215	0.128
Dark×35 °C × 24 h	− 1.963	0.215	0.14
Dark×35 °C × 48 h	− 2.309	0.215	0.099

The results from the fitted model show that the largest impact on pigmentation was from biomass, where the washed biomass resulted in approximately three times larger pigment production than the unwashed (0.368/0.11 = 3.34). The second largest main effect was from light with a two times increase in pigment production under light (0.309/0.131 = 2.36); thus, the change from dark to light condition in the post-treatment step and from unwashed to washed biomass results in a major increase in pigment production. No significant effects were found for a change from 24 to 48 h submerged fermentation and change in temperature from 25 to 35 °C. Generally, light has been shown to stimulate carotenoid synthesis in many fungi, related to the effect of oxygen radicals [6]. Increased carotenoid synthesis upon illumination on dark-grown cultures of *Neurospora crassa* and *Fusarium aquaeductuum* was reported by Rau and Rau-Hund [21]. Similar experiments with *N. crassa* have been reported by Zalokar [18] investigating the effect of light on pigment production in the fungal biomass. Comparable to this work, the maximum amount of pigments was achieved after exposing the mycelium pads to light and oxygen for 24 h (5 μg β-carotene and 65 μg γ-carotene/g of dry biomass). The author proposes light activation to be of an oxidative nature since illumination in the absence of oxygen failed to promote pigmentation [18]. Furthermore, improved carotene accumulation by *Blakeslea trispora* wild-type strain upon illumination was reported by Quiles-Rosillo [22]. Mycelia continuously grown on agar PDA medium in light for 4 days were compared to mycelia that have been grown in the dark before being illuminated, resulting in an improved carotene synthesis from 106 ± 5 to 352 ± 34 μg β-carotene/g dry biomass [22].

For two-factor interactions, significant effects were found for light×submerged fermentation time and temperature×biomass, with a positive effect for fermentation in light with 48 h submerged fermentation, and washed biomass at 35 °C. Together with the positive main effects for light and washed biomass, this shows a high pigment production for the combination of light, washed biomass, 35 °C, and 48 h submerged fermentation. Temperature was chosen as a factor since it is suspected to influence pigmentation as previous reports have highlighted temperature as an important factor regulating secondary metabolites. Studies have shown that different isolates of *Monascus* sp. resulted in a maximum pigment production at 30 °C [23].

The significant three-factor interaction light×temperature×submerged fermentation time was positive for pigment production in light, at 35 °C for 48 h, further improving the process conditions identified above. We can note the size of interaction effects is small compared to the main effects for light and biomass. Also, the highest predicted pigmentation according to the model, 1.4 mg β-carotene/g dry fungal biomass with a 95% confidence interval (0.771; 2.66), obtained under light, with washed fungal biomass, incubated at 35 °C and submerged fermentation for 48 h, can be compared with the original mean value of 1.3 mg β-carotene/g dry fungal biomass.

### Enhanced Conditions from Single Factors

#### Amount of Biomass and Harvesting Time

**Fig. 2:**
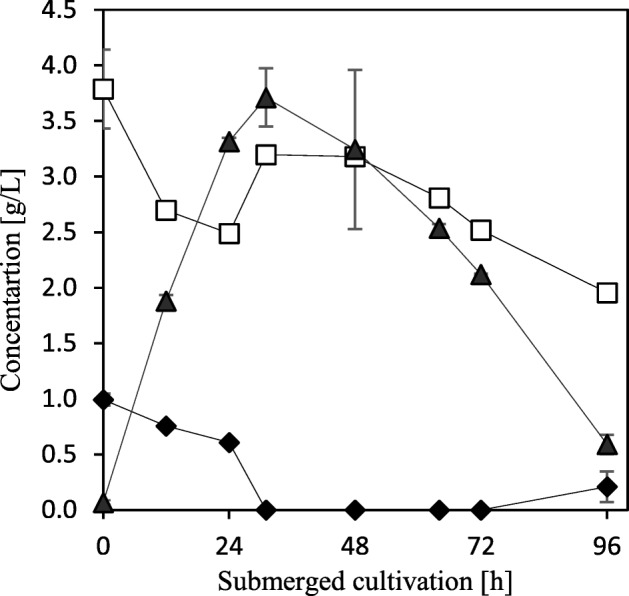
The concentration profiles of glucose (filled diamonds), other sugars (squares), and ethanol (filled triangles) in thin stillage over time in the bubble column reactor are also shown. The results are expressed as the mean ± standard deviation

Not only the pigment concentration but also the amount of fungal biomass produced in thin stillage is relevant for various applications such as for fish feed. Therefore, the amount of fungal biomass produced after 24 h and 48 h was investigated, as well as the harvested biomass potential, to produce pigments in the post-treatment step after different submerged fermentation times (24, 48, 72, and 96 h). The biomass concentration was measured at harvest as grams per liter thin stillage. The available nutrients in thin stillage were not fully consumed until 48 h fermentation had passed; consequently, a higher amount of fungal biomass (5.04 ± 0.1 compared to 3.98 ± 0.1 g/L) was obtained after 48 h cultivation compared to 24 h. Thin stillage is a complex medium in which saccharides and sugar polymers contain carbon sources which *N. intermedia* can use to produce biomass. The glucose and other sugars analyzed in thin stillage are low in comparison with the high amount of fungal biomass obtained at the end of fermentation (Fig. 2). The profiles of these sugars are a result of simultaneous hydrolysis and consumption until carbohydrates are depleted or until the fungus cannot, for some reason, hydrolyze more carbohydrates for consumption [3]. In addition, fungal biomass is being produced from other thin stillage components not analyzed in this study. More interestingly, the peak of biomass concentration in thin stillage correlated with the highest pigment concentration in the biomass after the post-treatment step. Biomass harvested after 24 h, i.e., during the exponential phase, resulted in a lower pigment production. Possibly, the change in metabolism from exponential to stationary phase affects pigment synthesis by *N. intermedia* in the post-treatment. Therefore, the harvested biomass’ ability to produce pigments in the post-treatment step was further studied in relation to the submerged fermentation time as the single factor, keeping all other parameters constant. The maximum pigment production, 1.03 ± 0.18 mg/g dry biomass, was obtained after 46 h of submerged fermentation in thin stillage. This was 15 h after glucose was not detectable and ethanol consumption had started, indicating that the maximum biomass production was reached within this time interval. The highest pigment production value obtained from the different time intervals can be compared to 0.67 ± 0.04 and 0.53 ± 0.19 mg/g dry biomass produced after 24 h and 72 h respectively. Under favorable nutritional conditions, acetyl-CoA is accumulated in the cells as a precursor of the tricarboxylic acid (TCA) cycle. However, acetyl-CoA is also the precursor of fatty acids and carotenoids [24]. One possible explanation for the higher pigment production from biomass harvested after 46 h than 24 h could be the higher accumulation of acetyl-CoA available to be synthesized to carotenoids in the post-treatment. A fermentation time over 46 h, in which glucose depletion happened, is speculated to result in less accumulated acetyl-CoA in the cells available for carotenoid synthesis. Comparable results were reported by Zalokar [18] cultivating *N. crassa* in submerged fermentation for 46 h to provide optimized conditions for pigmentation of the harvested pale mycelial pads in a later stage. Another study highlighted the vigorous growth of *N. crassa* in well-aerated, liquid cultures; nevertheless, pigmentation was limited to that portion of the mycelium growth which was exposed to the liquid surface or extended above it. Growth and uniform pigmentation were only obtained on a solidified agar medium incubated for 10–15 days [25].

The pale harvested biomass of *N. intermedia* showed no significant morphological changes between the samples at different harvesting times, which suggests that the processes involved in cell growth and morphology were probably not related to the biomass’ ability to produce pigments during post-treatment.

#### Biomass Moisture Content

Moisture content of the fungal biomass is suspected to be an important factor for post-treatment of pure fungal biomass. However, to our knowledge, no previous studies have investigated the effect of fungal biomass moisture content in a post-treatment step. The hypothesis is partly based on studies made on solid-state fermentation where the initial moisture of the substrate is one of the most important factors controlling secondary metabolite production [26]. The effect of the biomass moisture content on the production of pigments by *N. intermedia* in the post-treatment step was studied. Five different moisture contents were investigated as the sole variable ranging from 76 to 97%. The experiment demonstrated that the moisture content affected pigment production and could enhance pigmentation in the biomass at optimized conditions. A moisture content around 80% resulted in the highest pigment accumulation in the biomass (0.53 ± 0.05 mg/g dry biomass). A potential explanation is that a moisture content of 80% exposes the mycelium to sufficient light and air for the fungi to start producing pigments. A further increase in moisture content results in a layer of water on the mycelium that acts as a protection of the mycelium to oxygen which makes the response in the post-treatment step less effective. On the other hand, a far too low moisture content causes the fungus biomass to dry out, which results in a reduced metabolic activity and consequently pigment synthesis. Previous results for red pigment production by *Monascus purpureus* [23] suggest that lower pigmentation ability in submerged fermentation or very high initial moisture contents could be due to the restricted supply of oxygen and that a high initial moisture content promotes high glucose utilization that can inhibit pigment production. Another study highlighted the absence of pigments in *N. crassa* fungal biomass as long as the hypha were kept wet, and pigmentation was only induced when the conidia were formed in contact with air [18]. In the literature, no previous studies could be detected regarding different moisture contents that affect post-treatment of pure fungal biomass. However, the optimum moisture content for pigment production in solid- state fermentation varies extremely between species. Commonly, the optimum moisture content of the solid-state fermentation mode ranged from 22 to 60%; nonetheless, a moisture content requirement as high as 96% has also been reported [6].

#### Thickness of the Biomass Layer

**Fig. 3:**
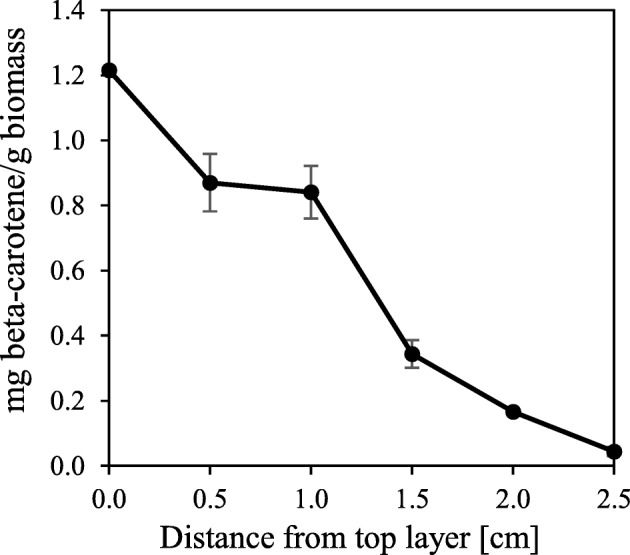
Pigment production in *N. intermedia* biomass after incubation in a glass beaker for 48 h in 95% Rh, at 35 °C under light. The *x*-axis represents the distance from the top height of the fungal biomass. The results are expressed as the mean ± standard deviation

To examine the post-treatment method’s potential in larger scale, the maximum thickness of the biomass layer that can be practiced while keeping a homogeneous pigmentation needs to be addressed. The maximum height of the fungal biomass applied while keeping a homogenous pigmentation in the biomass was investigated. In 95% Rh, 35 °C under light resulted in pigmentation to a depth of 1.5 ± 0.3 cm, after which pigmentation was close to zero (Fig. 3). The experiment was performed both with and without exposing the sides of the biomass to light. No significant difference was detected, however. This indicates that a combination of light and oxygen is required for the pigmentation. A previous study that supports the importance of oxygen has been reported for production of pigments by *N. crassa*. The results from the study demonstrate an increase in *al-1* and *al-2* (genes involved in synthesis of carotenoids) transcript levels 16 h after transferring submerged grown mycelia to open air, coinciding with the formation of pigments [27]. Another study points out the importance of light and need of oxygen in the formation of pigments after it was observed that not all parts of the *Neurospora* mycelium was equally colored after the mycelium was left on the petri dish [18]. In this study, light seems to work as a trigger for pigmentation but the ability for oxygen to penetrate through the biomass is critical to obtain homogeneously pigmented fungal biomass. To increase the thickness further while keeping the biomass equally colored, one possible solution can be to force aeration through the bed of biomass. However, taking into account the present industrial fermentation processes for solid biomass, this setup can be very challenging and requires further comprehensive and detailed studies. *Monascus ruber* was cultivated in a packed bed solid state for pigment production. High pigment production was obtained by forced humidified aeration through the bed up to 0.5 L/min, after which pigment production decreased due to water loss from the bed [28].

#### Light Exposure of the Fungal Biomass in Post-treatment

The effect of light exposure time in the post-treatment of *N. intermedia* biomass was also studied at 35 °C in 95% relative humidity. Light exposure for only 1 min was enough to stimulate pigmentation, producing 0.67 ± 0.08 mg/g dry biomass, and the same amount was obtained after 1 h exposure time. Nonetheless, a higher amount of pigment was still observed in samples treated with continuous light exposure for 24 h, at 0.53 ± 0.19 mg/g dry biomass. Regarding light as a trigger, the fungal biomass of *N. crassa* exposed to light for 1 min has been reported to be sufficient to stimulate full pigment production in the presence of oxygen whereas oxygen needed to be present to achieve full pigmentation [18]. Most of the pigments have been reported to be concentrated in the conidia and in the conidia-bearing hyphae [18]. In this study, it was observed that a high degree of moisture content (approximately > 90%) resulted in most pigments being produced in the conidia, whereas a lower moisture content resulted in pigmented mycelium without the dusty pigment powder produced in the conidia. Another interesting study stated that cultures containing both mycelia and conidia produced visible pigments in the conidia even in the dark, whereas the mycelium is usually colorless [25]. In this study, the same results were not observed in dark-grown cultures during post-treatment. The authors went on experimenting with blue, red, and white light. Red light had no stimulatory effect on pigment production whereas exposure to blue and white light resulted in a similar color change. The color change upon exposure to white light was mostly noticed within the mycelium [25]. The source of light and the intensity of the light are important factors for large-scale applications and are therefore suggested to be investigated in future studies.

### Protein Content of Fungal Biomass

Since the pigmented biomass has potential applications in the feed industry, the protein content (% *w*/*w*) of the washed biomass before and after post-treatment was analyzed as an important characteristic for feed. The crude protein contents were not found to be statistically different at the conditions examined (*p* = 0.13). However, the possible trend observed in the results indicated the protein content to be highest before post-treatment (52.09 ± 0.54). The slight decrease in protein content after post-treatment to 51.53 ± 0.01 and 50.18 ± 1.00 for 24 and 48 h, respectively, might be related to changes in the catabolic state in the fungi caused by the absence of a carbon source. The protein values in this paper are in agreement with Ferreira [3] who reported a protein content of 50% from the cultivation of *N. intermedia* in thin stillage where all nine amino acids essential to humans were presented.

### Pigment Production by *N. intermedia* in the Pilot Bioreactor

The method was also scaled up from primary shake-flask experiments to pilot scale using 26-L and 1-m^3^ bioreactors. The biomass was easy to separate from the medium. Post-treatment of the pale biomass for 24 h with controlled moisture and temperature obtained 1.02 ± 0.17 mg of fungal pigments/g of dry biomass. Further scale-up to a 1-m^3^ airlift bioreactor resulted in 4.25 ± 0.26 kg biomass after 48 h. The biomass was successfully pigmented in the post-treatment steps although some modifications were made. The biomass was spread with a thickness layer of ca 1.5 cm and left at room temperature under normal light for 24 h. Within 3 days, 0.83 ± 0.48 mg β-carotene/g dry biomass was obtained. Since 2000, the fungus *B. trispora* is the single microorganism used in industrial scale for the production of β-carotene in a conventional stirred tank reactor using synthetic medium. The fungus can produce 13–19 mg β-carotene per g of dry biomass after 5–8 days [29]. To make the production of pigments and other valuable components from the cultivation of ascomycetes competitive to that of synthetic ones or other natural sources such as plants and fruits, the synthesis needs to be time efficient and made from a simple fermentation system that can easily be scaled up [1, 10, 30]. The possibility of treating large amounts of fungal biomass presented in this work indicates the methods’ potential for large-scale production of pigmented fungal biomass.

### Implications of the Post-treatment Strategy

The potentiality of increasing valuable secondary metabolite production reinforces the already-high-value biomass from edible filamentous fungi. In addition, utilizing edible strains already employed in preparing fermented foods for human consumption significantly reduces the amount of testing required prior to a full-scale process for feed production. *N. intermedia* has previously been reported to be a good candidate among the Ascomycetes for biomass (ca. 4 g/L) and ethanol (ca. 5 g/L) production from thin stillage [3]. As stated by Ferreira [3], *N. intermedia* has potential for inclusion in the industrial process of ethanol via production of additional ethanol and fungal biomass. The post-treatment method in this work is an efficient method to further boost the profitability by converting ethanol by-products into pigmented biomass which can be considered for animal feed applications. In addition, the remaining liquid fraction of thin stillage after submerged cultivation, containing ethanol (3.7 ± 0.3 g/L) and potentially lower organic loads, can be integrated into the current first-generation ethanol plant process to improve the industrial process [3]. Overall, post-treatment of edible filamentous fungi cultivated on waste streams has the potential to add functionality to biomass.

## Conclusion

A post-treatment method to achieve a high amount of fungal biomass with enhanced secondary metabolite production is presented. The results obtained by combining submerged fermentation with post-treatment illustrate the high potential for pigment production by *N. intermedia*. Submerged cultures grown in thin stillage remained colorless and pigmentation started during exposure to light and oxygen in a post-treatment step. In optimized conditions of biomass moisture (80%), light, temperature (35 °C), and fermentation time (46 h), *N. intermedia* produced 1.4 ± 0.3 mg pigments/g dry biomass. The new method opens an avenue for industrial application of pigments and potentially other metabolites on waste products as valuable products.

## References

[1] Ferreira JA, Mahboubi A, Lennartsson PR, Taherzadeh MJ (2016). Waste biorefineries using filamentous ascomycetes fungi: present status and future prospects. Bioresource Technology.

[2] Cherubini, F. 2010. The biorefinery concept: using biomass instead of oil for producing energy andchemicals. *Energy conversion and management, 51*(7), 1412-1421.

[3] Ferreira JA, Lennartsson PR, Taherzadeh MJ (2015). Production of ethanol and biomass from thin stillage by Neurospora intermedia: a pilot study for process diversification. Engineering in Life Sciences.

[4] Subramaniyam R, Vimala R (2012). Solid state and submerged fermentation for the production of bioactive substances: a comparative study. International Journal of Natural Sciences.

[5] Hölker U, Höfer M, Lenz J (2004). Biotechnological advantages of laboratory-scale solid-state fermentation with fungi. Applied Microbiology and Biotechnology.

[6] Ogbonna CN (2016). Production of food colourants by filamentous fungi. African Journal of Microbiology Research.

[7] Huang T, Wang M, Shi K, Chen G, Tian X, Wu Z (2017). Metabolism and secretion of yellow pigment under high glucose stress with Monascus ruber. AMB Express.

[8] Aruldass CA, Dufossé L, Ahmad WA (2018). Current perspective of yellowish-orange pigments from microorganisms-a review. Journal of Cleaner Production.

[9] Silva TP, Paixão SM, Alves L (2016). Ability of Gordonia alkanivorans strain 1B for high added value carotenoids production. RSC Advances.

[10] Pagano MC, Dhar PP, Gupta VK, Mach RL, Sreenivasaprasad S (2015). Fungal pigments: an overview. Fungal bio-molecules: sources, applications and recent developments.

[11] Priatni S (2014). Review: Potential production of carotenoids from Neurospora. Nusantara Biocenter.

[12] Zalokar M (1957). Variations in the production of carotenoids in Neurospora. Archives of Biochemistry and Biophysics.

[13] Nuraini S, Latif SA (2009). Improving the quality of tapioca by product through fermentation by Neurospora crassa to produce $ carotene rich feed. Pakistan Journal of Nutrition.

[14] Ferreira JA, Lennartsson PR, Taherzadeh MJ (2014). Production of ethanol and biomass from thin stillage using food-grade Zygomycetes and Ascomycetes filamentous fungi. Energies.

[15] Gmoser R (2018). Pigment production by the edible filamentous fungus Neurospora intermedia. Fermentation.

[16] Singgih M (2015). Carotenogenesis study of Neurospora intermedia N-1 in liquid substrate fermentation. Journal of Chemical and Pharmaceutical Research.

[17] Sowti Khiabani M, Hamidi Esfahani Z, Azizi MH, Sahari MA (2011). Effective factors on stimulate and stability of synthesised carotenoid by Neurospora intermedia. Nutrition & Food Science.

[18] Zalokar M (1954). Studies on biosynthesis of carotenoids in Neurospora crassa. Archives of Biochemistry and Biophysics.

[19] Dean, A., Voss, D., Draguljić, D. (1999). Checking model assumptions. In *Design and analysis of experiments* (Vol. 1, pp. 103–137). Cham: Springer.

[20] Souza Filho PF, Zamani A, Taherzadeh MJ (2017). Production of edible fungi from potato protein liquor (PPL) in airlift bioreactor. Fermentation.

[21] Rau W, Rau-Hund A (1977). Light-dependent carotenoid synthesis. X. Lag-phase after a second illumination period in Fusarium aquaeductuum and Neurospora crassa. Planta.

[22] Quiles-Rosillo MD, Ruiz-Vázquez RM, Torres-Martínez S, Garre V (2005). Light induction of the carotenoid biosynthesis pathway in Blakeslea trispora. Fungal Genetics and Biology.

[23] Babitha S, Soccol CR, Pandey A (2007). Solid-state fermentation for the production of Monascus pigments from jackfruit seed. Bioresource Technology.

[24] He Z (2017). β-Carotene production promoted by ethylene in *Blakeslea trispora* and the mechanism involved in metabolic responses. Process Biochemistry.

[25] Haxo F (1949). Studies on the carotenoid pigments of Neurospora. 1. Composition of the pigment. Archives of Biochemistry.

[26] Johns MR, Stuart DM (1991). Production of pigments by Monascus purpureus in solid culture. Journal of Industrial Microbiology & Biotechnology.

[27] Avalos, J., Nordzieke, S., Parra, O., Pardo-Medina, J., Limón, M.C. (2017). Carotenoid Production by Filamentous Fungi and Yeasts. In: Sibirny A. (eds) *Biotechnology of yeasts and filamentous fungi* (pp. 225–279). 10.1007/978-3-319-58829-2_8.

[28] Said FM, Chisti Y, Brooks J (2010). The effects of forced aeration and initial moisture level on red pigment and biomass production by Monascus ruber in packed bed solid state fermentation. International Journal of Environmental Science and Development.

[29] Ribeiro BD, Barreto DW, Coelho MAZ (2011). Technological aspects of β-carotene production. Food and Bioprocess Technology.

[30] Velmurugan P, Lee YH, Venil CK, Lakshmanaperumalsamy P, Chae JC, Oh BT (2010). Effect of light on growth, intracellular and extracellular pigment production by five pigment-producing filamentous fungi in synthetic medium. Journal of Bioscience and Bioengineering.

